# Ang-(1–7)/MasR axis promotes functional recovery after spinal cord injury by regulating microglia/macrophage polarization

**DOI:** 10.1186/s13578-023-00967-y

**Published:** 2023-02-04

**Authors:** Guangjin Gu, Bin Zhu, Jie Ren, Xiaomeng Song, Baoyou Fan, Han Ding, Jun Shang, Heng Wu, Junjin Li, Hongda Wang, Jinze Li, Zhijian Wei, Shiqing Feng

**Affiliations:** 1 Tianjin Key Laboratory of Spine and Spinal Cord Injury, Department of Orthopedics, National Spinal Cord Injury International Cooperation Base, Tianjin Medical University General Hospital, Anshan Road 154, Heping District, Tianjin, 300052 China; 2 Tianjin Key Laboratory of Lung Cancer Metastasis and Tumor Microenvironment, Tianjin Lung Cancer Institute, Tianjin Medical University General Hospital, Tianjin, China; 3 Department of Orthopaedics, Qilu Hospital of Shandong University, Shandong University Centre for Orthopaedics, Advanced Medical Research Institute, Shandong University, Jinan, Shandong China

**Keywords:** Spinal cord injury, Ang-(1–7), MasR, Mocroglia, Macrophages, Inflammation

## Abstract

**Background:**

Inflammatory response is an essential part of secondary injury after spinal cord injury (SCI). During this period, the injury may be exacerbated through the release of a large number of inflammatory factors and the polarization of infiltrating macrophages and microglia towards M1. Ang-(1–7), mainly generated by Ang II via angiotensin-converting enzyme 2 (ACE2), can specifically bind to the G protein-coupled receptor Mas (MasR) and plays an important role in regulating inflammation and alleviating oxidative stress.

**Methods:**

We aimed to investigate whether activating the Ang-(1–7)/MasR axis in rats after SCI can regulate local neuroinflammation to achieve functional recovery and obtain its potential mechanism. MasR expression of bone marrow-derived macrophages was determined by Western blot. Immunofluorescence, Western blot, Flow cytometry, and RT-qPCR were applied to evaluate the polarization of Ang-(1–7) on macrophages and the regulation of inflammatory cytokines. Previous evaluation of the spinal cord and bladder after SCI was conducted by hematoxylin–eosin staining, Basso, Beattie, and Bresnahan (BBB) score, inclined plate test, electrophysiology, and catwalk were used to evaluate the functional recovery of rats.

**Results:**

MasR expression increased in macrophages under inflammatory conditions and further elevated after Ang-(1–7) treatment. Both in vivo and in vitro results confirmed that Ang-(1–7) could regulate the expression of inflammatory cytokines by down-regulating proinflammatory cytokines and up-regulating anti-inflammatory cytokines, and bias the polarization direction of microglia/macrophages to M2 phenotypic. After SCI, Ang-(1–7) administration in situ led to better histological and functional recovery in rats, and this recovery at least partly involved the TLR4/NF-κB signaling pathway.

**Conclusion:**

As shown in our data, activating Ang-(1–7)/MasR axis can effectively improve the inflammatory microenvironment after spinal cord injury, promote the polarization of microglia/macrophages towards the M2 phenotype, and finally support the recovery of motor function. Therefore, we suggest using Ang-(1–7) as a feasible treatment strategy for spinal cord injury to minimize the negative consequences of the inflammatory microenvironment after spinal cord injury.

## Introduction

Spinal cord injury, as a permanent injury, brings heavy social and financial burdens to patients and their families, Statistics have shown that the prevalence rate of spinal cord injury in the world has surged from 236 to 1298 cases per million people every year over the past three decades [1]. Following primary injuries such as strike, compression, and torsion, secondary injuries caused by inflammation, oxidative stress, ischemia, and hypoxia in the spinal cord microenvironment can aggravate local neuron death and demyelination [2]. As an essential element of secondary injury, the inflammatory reaction can induce in situ microglia activation, macrophage infiltration, and glial scar formation [3]. In addition, the early inflammatory reaction after traumatic spinal cord injury suggests the necessity of intervention after primary injury in a timely manner [4, 5]. Therefore, controlling local inflammation may be one of the therapeutic strategies to improve injury and promote the recovery of motor function.

Mechanically, 6–12 h after primary spinal cord injury, microglia cells near the injury site are activated sequentially and migrate to the injury site, releasing inflammatory cytokines [6]. A peak of macrophage infiltration occurred 7 days after spinal cord injury. Macrophage acts similarly to microglia (specific monocytes of the central nervous system) under inflammatory conditions and can polarize into M1 macrophages under the action of the T helper 1 (Th1) cytokine (such as interferon-γ (IFN-γ), tumor necrosis factor-α (TNF-α) and lipopolysaccharide(LPS), then it produces toxic mediators and pro-inflammatory cytokines, and at the same time enhances its antigen presenting capacity and phagocytosis capacity, which leads to further destruction of tissues and polarization of more macrophages to M1 [7]. M1 macrophages express high levels of inducible nitric oxide synthase (iNOS), CD86, and CD16/CD32, and its characteristic cytokines include interleukin (IL)-6, IL-12, and IL-23 [8]. In comparison, M2 macrophages can be induced by TH2 cytokines (such as IL-4 and IL-10). The classical cell markers are arginase 1 (Arg-1), CD163, and CD206, and the characteristic cytokines include IL-4 and IL-10 [9]. M2 macrophages can resist inflammation and promote tissue regeneration after spinal cord injury. Similarly, microglia also have such classification, including M1-like microglia and M2-like microglia [10]. Shuhei Kobashi et al. have found that transplanting M2-like microglia into spinal cord injury model mice can achieve histological and functional recovery [11]. Jiaxing Wang et al. also reported that the use of bone marrow-derived M2 macrophage exosomes rich in microRNA-421-3p can target mammalian rapamycin targets (mTOR) to reduce neuronal apoptosis, improve nerve tissue injury and promote functional recovery by increasing autophagy [12]. References: As per pubmed findings, citation details [*<specify missing detail>*] for Reference [__] have been inserted. Kindly check and confirm the inserted details.

Angiotensin-(1–7) (Ang-(1–7), mainly produced by ACE2-dependent lysis of angiotensin II (Ang II), can bind to G protein-coupled receptor Mas receptor (MasR) and antagonize ACE/Ang II/AT1R axis, which has long been regarded as harmful classical axis [13]. A large number of studies have suggested that, Ang-(1–7)/MasR axis, the protective arm of the renin-angiotensin system (RAS), can not only regulate cardiovascular system and body fluid balance but also resist inflammation [14–17]. Besides, previous studies have shown that, in addition to the classical circulating RAS, the second RAS (local or tissue RAS) can be founded in many tissues such as the brain, and local RAS in the brain participates in the activation process of microglia [18, 19]. Zachary C et al. showed that subcutaneous injection of Ang-(1–7) can effectively promote the recovery of traumatic brain injury by reducing brain trauma area, decreasing glial cell reactivity, and promoting angiogenesis [20]. Similarly, Robert W et al. agreed that Ang-(1–7) has a protective effect on the brain and can alleviate cerebral ischemia–reperfusion injury by weakening the up-regulation of iNOS mRNA and reducing the expression of pro-inflammatory cytokines in ischemic stroke [21].

At present, the regulation of inflammation after spinal cord injury involves drugs, biomaterials, gene regulation, etc.… However, Ang-(1–7)/MasR axis is a regulatory system produced locally in tissues, and there is no research report targeting the repair of spinal cord injury. As demonstrated in the study, the local Ang-(1–7)/MasR axis is involved in repairing the inflammatory pathway of traumatic spinal cord injury, which paves the way for mitigating spinal cord injury and improving neurological outcomes.

## Methods

### Macrophage cultures and treatment

Bone marrow-derived macrophages (BMDMs) were extracted from the femurs and tibias of rats [22]. Primary cells were cultured in DF-12 medium mixed with 20 ng/ml M-CSF (HY-P7247, MCE), 10% Fetal Bovine Serum and 1% penicillin/streptomycin in a humidified incubator at 37℃ with 5% CO_2_.

On day 7, macrophages were divided into 5 groups: control group, LPS group, Ang-(1–7) group, LPS + Ang-(1–7) group, and LPS + Ang-(1–7) + A779 group. A779, as a Mas receptor antagonist, was pretreated 30 min before LPS and Ang-(1–7) intervention. Macrophages were treated with LPS (100 ng/ mL, L3025, Sigma), Ang-(1–7) (10^−6^ mol/L, HY-12403, MCE) and A779 (10^−5^ mol/L, HY-P0216, MCE) for 24 h, respectively.

### Animals

200 female Wistar rats, with each weighing 200-220 g on average, were purchased from Beijing Vital River Laboratory Animal Technology Co., Ltd (Beijing, China, Permission Number: SCXK (Jing)—2016—0011). All rats were housed under controlled environmental conditions (temperature 22–24 ℃, humidity 60–80%) on a 12 h light–dark cycle. The animal study was approved by the Ethics Committee of the Institute of Radiation Medicine, Chinese Academy of Medical Sciences (Tianjin, China, Approval number: IRM-DWLL-2021179).

### Contusion model of spinal cord and drug treatment

In accordance with the previous reports [23], the spinal cord injury model was established by the following procedure: the rats were anesthetized by inhalation of isoflurane. The skin of the back was incised and the paraspinal muscles separated, exposing the spinal cord after a T10 laminectomy. The spinal cord was struck with a 10 g x 25 mm free-falling strike. After a successful strike, there will be a rat tail swing and hind limb spasm. Different concentrations of Ang-(1–7) were administered immediately by intrathecal injection (10 μL; 100 ng, 500 ng, 1000 ng), and A779 (10 μL; 500 ng) was injected half an hour earlier than Ang-(1–7). As controls, the SCI and Sham groups were injected intrathecally with equal doses of PBS. Then the muscles and skin were sutured and placed on a 40 ℃ thermostatic pad for resuscitation. The rats were transferred to cages after adequate rest. Urinary retention was alleviated by bladder compression twice a day until urination function was restored. Spinal cord shock was excluded in all Sham groups, and other operations were the same as those in the injured group.

### RNA extraction and quantitative reverse transcription PCR

Total RNA was extracted from primary macrophages and spinal cord tissue using an extraction buffer (Trizol/phenol/chloroform). Similarly, cDNA reverse transcription was performed with HIFIScript gDNA Removal RT MasterMix (CWBio, CW 2020) and RT-qPCR was performed using UltraSYBR Mixture (CWBio, CW 0957). The primers employed in the experiment are listed in the following table 1.

### Cytosolic and nuclear protein extraction and isolation

Each group of proteins was extracted from macrophages at 24 h of corresponding treatment and spinal cord tissue at 3 days after surgery.

**Table 1:** Sequences of primers for real-time quantitative polymerase chain reaction

Gene	Forward primer sequence (5′–3′)	Reverse primer sequence (5′–3′)
*IL-1β*	GCTTCCTTGTGCAAGTGTCT	TCTGGACAGCCCAAGTCAAG
*TNF-α*	ATGGGCTCCCTCTCATCAGT	GCTTGGTGGTTTGCTACGAC
*IL-4*	GTACCGGGAACGGTATCCAC	TGGTGTTCCTTGTTGCCGTA
*IL-10*	CGACGCTGTCATCGATTTCTC	CAGTAGATGCCGGGTGGTTC
*GAPDH*	AGTGCCAGCCTCGTCTCATA	AGCCCTGTATTCCGTCTCCT

The Nuclear and Cytoplasmic Protein Extraction Kit (Beyotime, P0027) was used in this study to extract the nuclear and cytosolic protein. Protease and phosphatase inhibitor cocktail (Beyotime, P1046) was used to inhibit phosphorylation and degradation of extracted proteins. All operations are conducted in accordance with the manufacturer’s protocol.

### Western blot analysis

The protein was then premixed with SDS-PAGE (CW0027S, CWBIO) in a 4:1 ratio and heated at 100 ℃ for 10 min. The protein samples were separated and transferred to *polyvinylidene fluoride* (PVDF) membrane. Then, the PVDF membrane was blocked with 5% nonfat milk for 1 h and incubated overnight with a primary antibody. Subsequently, the PVDF membranes were incubated with corresponding secondary antibodies at room temperature for 1 h and ECL Kit Chemiluminescence (S6008M, US EVERBRIGHT) was applied to visualize the immunoblots. Finally, ImageJ software was used for protein analysis.

The primary antibodies used in Western blot are as follows: anti-mas1 (1:1000, Proteintech, 20080–1-ap), anti-IL-1β (1:2000, Proteintech, 26,048–1-AP), anti-TNF-α (1:1000, Proteintech, 60291–1-Ig), anti iNOS (1:1000, Abcam, ab283655), anti-Arg-1 (1:1000, Abcam, ab283574), anti-p65(1:1000, Abcam, ab16502), anti-p65 (phospho S536) (1:1000, Abcam, ab86299), anti-TLR4(1:1000, Proteintech, 19811–1-AP), anti-IκB(1:2000, Proteintech, 10268–1-AP), anti-GAPDH (1:1000, Abcam, ab18602), anti-βactin (1:20000, Proteintech, 66009–1-Ig), anti-vinculin (1:10000, Proteintech, 66305–1-Ig).

### Immunofluorescence staining

#### Cellular immunofluorescence

The cell culture medium was sucked out and cleaned with PBS. BMDMs were fixed with 4% PFA for 15 min, then permeabilized with 0.25% Triton x-100 and sealed with 5% BSA at room temperature for 1 h. The slides were incubated overnight with the primary antibodies at 4 ℃ and washed with PBST three times for 10 min each time. Then, the slides were incubated with secondary antibody for 1 h at room temperature in the dark. Finally, the slides were sealed with an anti-fluorescence quencher containing DAPI.

#### Tissue immunofluorescence

The Rats were anesthetized with pentobarbital sodium 3 days and 28 days after surgery and then perfused with PBS and 4% paraformaldehyde (PFA) at 4 ℃ through the heart. The spinal cord injury segments of rats were taken and fixed in 4% PFA at 4 ℃ overnight, and then transferred to 15% and 30% sucrose solution and dehydrated for 3 days. The tissue was embedded and then frozen at −80 ℃. The sample was sliced into 10 μm thickness. 0.25%Triton X-100 and 5% bovine serum albumin (BSA) were used to penetrate and seal tissue slices for 1 h. Next, the spinal cord sections were incubated overnight with primary antibodies at 4 ℃ and washed with TBST three times for 10 min each time. Then, the secondary antibody was incubated at room temperature in the dark for 1 h and washed with TBST for 3 times, 10 min for each time.

Images of each slide/section were randomly captured under LSM900 laser confocal super-resolution microscope and the fluorescence intensity of images of each experimental group was analyzed using *Image J* software (NIH, United States). The primary antibody used in the experiment is as follows: anti-mas1 (1:20, Proteintech, 20,080–1-ap), anti-F4/80 (1:100, Abcam, ab16911), anti-CD11b (1:500, Abcam, ab133357), anti iNOS (1:100, Abcam, ab283655), anti-Arg-1 (1:500, Abcam, ab283574), anti-Iba1 (1:100, abcam, ab283319), anti-CD206(1:500, Abcam, ab64693), anti-CD68(1:100, Abcam, ab283654), anti-GFAP (1:500, Abcam, ab53554), anti-NF200(1:500, abcam, ab8135).

#### Flow cytometry

The treated macrophages were digested into cell suspensions using 0.25% Trypsin–EDTA (Gbico, 25200072) and filtered through a 70 µm cell strainer (FALCON, 352350) to produce a single-cell suspension. Cells were incubated with surface markers against pre-conjugated antibodies PE-labeled Anti-CD86 Antibody (Biolegend, 200308), FitC-labeled anti-CD11b Antibody (Abcam, 184370) for 30 min at 4 ℃. Cells were then permeabilized for intracellular staining with Cyto-Fast Fix/Perm Buffer Set (Biolenged, 426803). Subsequently, cells were incubated with CD206 Antibody (Santa Cruz, sc-58986) for 40 min at room temperature in dark. Secondary antibodies APC (Abcam, ab150167) and FITC (Abcam, ab6717) were used in cells. 0.4% formaldehyde solution was used to fixed cells for detection. The samples were collected using BD FACS Diva software (BD Biosciences) on a BD LSRFortessa high-end analytical flow cytometer, and FlowJo software (TreeStar, Ashland, OR) was used for compensation and data analysis.

#### HE staining

After the Rats were anesthetized and perfused, the spinal cord and bladder were removed and fixed in 4% paraformaldehyde for 48 h. Following gradient alcohol dehydration, cleared in xylene and finally embedded in paraffin, 5 μm-thick tissue section slices were pasted on slides and stained with hematoxylin–eosin (G1120, Solarbio). HE staining is operated under the guidance of the user's manual. Finally, the images were obtained by a bright-field microscope (OLYMPUS, JAPAN, TH4-200).

#### Behavioral assessment

##### BBB score

The Basso, Beattie, and Bresnahan (BBB) scales were used to measure the functional recovery degree of the hind limbs after spinal cord injury in Rats by two uninformed evaluators. The scale, ranging from 0 to 21 points, assessed the number of joint movements, landing and loading on the balls of the feet, front and rear limb coordination, paw orientation, trunk movement stability, and tail elevation.

##### Inclined plane test

The rats were placed on a rectangular board, which was secured with a rubber pad. The plate can be tilted and the angle can be measured. The longitudinal axis of the rats’ body was placed parallel to the longitudinal axis of the inclined plane, and the rats’ head was raised to the side of the inclined plate. The angle of the inclined board was changed by 5° each time, and the maximum angle of the board when the rat could maintain its initial posture for 5 s was taken as the measurement value. Each animal was measured three times and its average value was taken.

##### Catwalk

Catwalk-assisted gait analysis was performed as described in other reports [24]. Briefly, rats were put on a horizontal glass track equipped with a standard charge-coupled device (CCD) camera for a certain period and relevant data were recorded to evaluate the motion coordination, such as regularity index (RI) and stride length. RI represents the ratio between actual and normal coordination of rat limbs. During the movement, the RI value increases with the number of correct gait sequences. Stride Length, a mobility analysis parameter, decreased significantly after spinal cord contusion. Baseline data were collected 3 days before surgery and the rats unable pass the training were excluded.

##### Electrophysiological

Electrical activity was assessed 4 weeks after SCI. In brief, each group of rats was anesthetized and their electrical activity was assessed by using an electrophysiological device (YRKJ-G2008; Zhuhai Yiruikeji Co, Ltd, Guangdong, China). Bipolar stimulation electrodes were embedded behind the head and the neck and recording electrodes were placed in the gastrocnemius muscle of the lower extremity to record and compare the peak amplitude and latency of motor evoked potentials (MEP) of rats in each group.

### Cavity area calculation

According to the ruler marked by the microscope in the image, image J software was used to convert pixels and Standard unit of length, and then the syringomyelia region was traced, and the corresponding cavity area was obtained by the software area algorithm.

### Statistical analysis

Numerical data from at least three independent experiments were analyzed by one-way ANOVA and Tukey's Post Hoc test and presented in mean ± SD. P < 0.05 was considered significant. Between-group differences in BBB scores and inclined plane test results were analyzed using repeated measurement two-way mixed ANOVA, followed by Bonferroni’s test. GraphPad Prism (Graph Pad Software, San Diego, CA, USA) was used to analyze data according to the previous description [22].

## Results

### Ang-(1–7) significantly up-regulated MasR expression in inflammatory induced macrophages

**Fig. 1:**
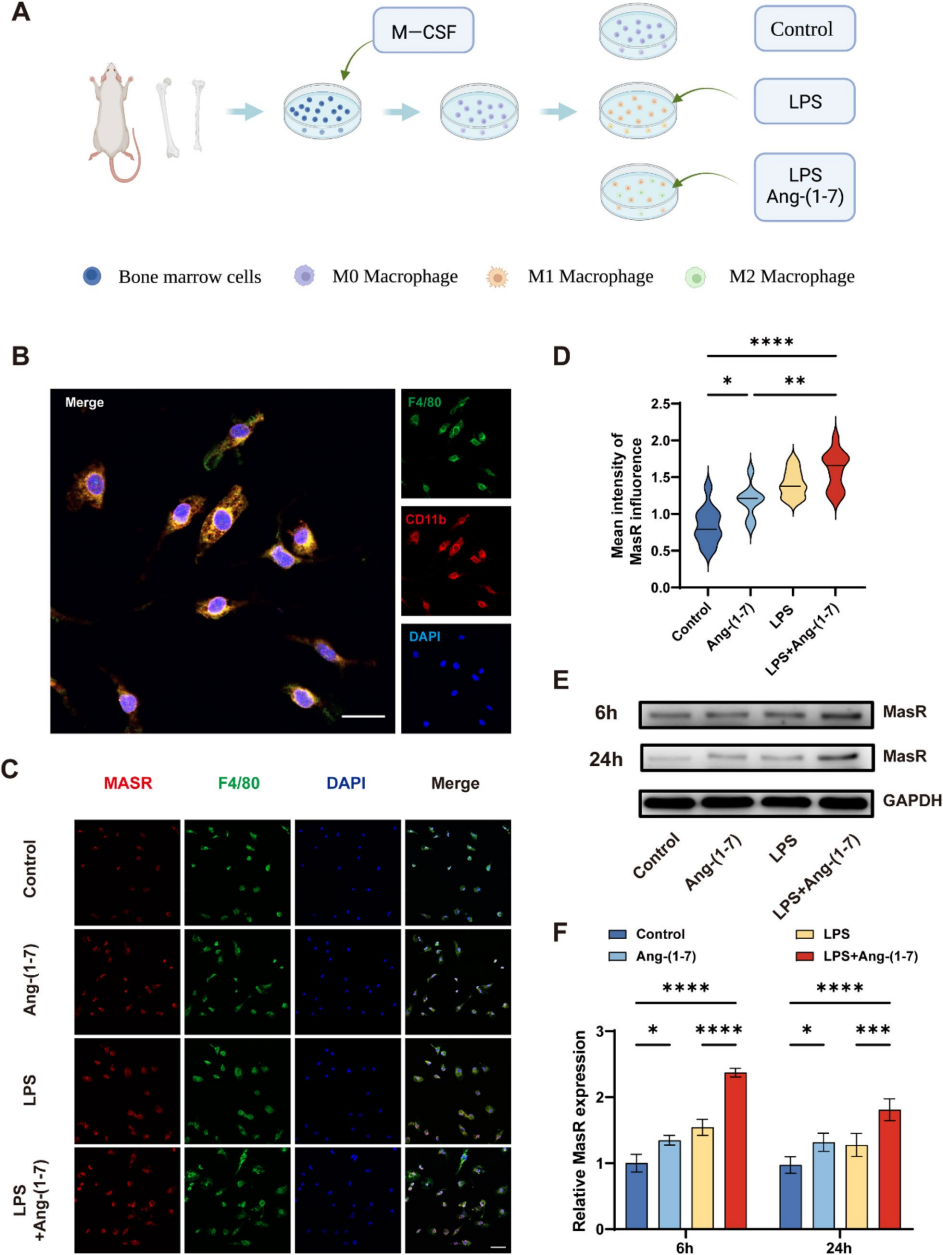
Expression changes of MasR in macrophages in each group after 24 h. **a** Schematic diagram of macrophages extracted and induced from rat femur and tibia. **b** Double-staining for F4/80 (green)/ CD11b (red) in macrophages from each group (scale bar: 20 μm). **c–d** Double-staining for F4/80 (green)/ MasR (red) in macrophages from each group (scale bar: 50 μm, n = 5). **e–f** Representative Western blots and quantification of MasR and GAPDH expression in each group of macrophages treated for 6 h and 24 h. (n = 3). data are presented as mean ± SD; *P < 0.05, **P < 0.01, ***P < 0.001, and ****P < 0.0001. In **c-d**, one-way ANOVA followed by Tukey’s Post Hoc test. In **e–f**, two-way ANOVA followed by Bonferroni’s post hoc tests

Bone marrow-derived macrophages were extracted from the femur and tibia of Wistar rats (Fig. 1a). F4/80 and CD11b were used as specific markers of macrophages, and immunofluorescence staining was applied to identify the extracted primary macrophages (Fig. 1b). The expression of MasR was analyzed by immunofluorescence staining, and the fluorescence intensity of macrophages in LPS and Ang-(1–7) groups was higher than that in the control group, and the fluorescence intensity of macrophages in LPS + Ang-(1–7) group was the highest (Fig. 1c, d). To further verify this phenomenon, Western blot analysis was performed to investigate the expression of MasR in macrophages induced by Ang-(1–7), LPS, and LPS + Ang-(1–7) for 6 h and 24 h. Compared with the control group, the expression of MasR was up-regulated in both Ang-(1–7) and LPS groups. The up-regulated expression was more obvious in LPS group than Ang-(1–7) group within 6 h, but the result was the opposite at 24 h. It is worth noting that the expression of MasR in the LPS + Ang-(1–7) group was the highest, about twice of that in the control group (Fig. 1e, f). Our results show that the expression of the MasR in macrophages under inflammatory conditions increases after being treated with Ang-(1–7), which indicates that Ang-(1–7) can play a larger role in macrophages expressing MasR under inflammatory conditions.

### Ang-(1–7) regulates the polarization of macrophages under inflammatory conditions

**Fig. 2:**
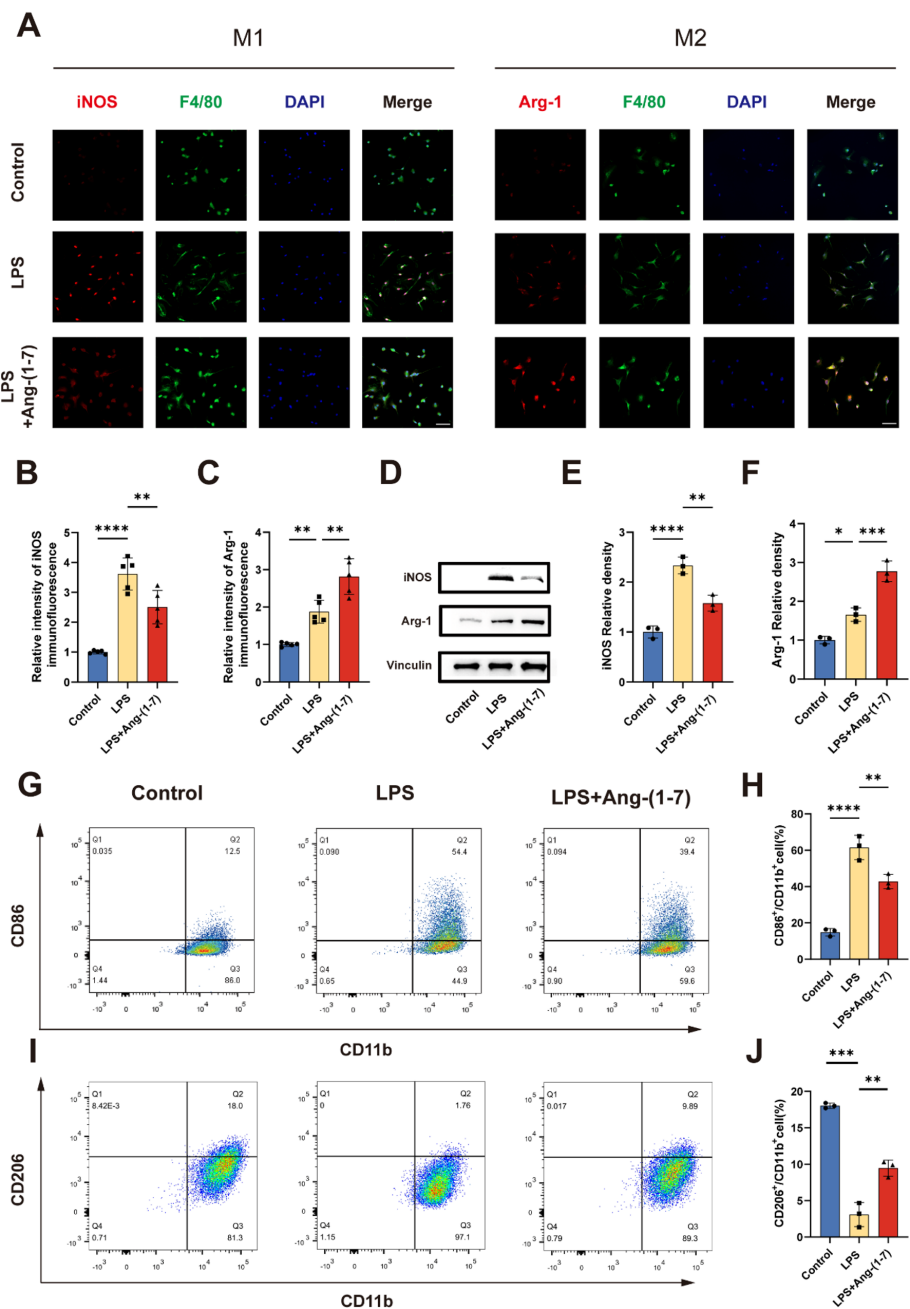
Ang-(1–7) regulates the polarization of macrophages towards the M2 phenotype. **a–c** Double-staining for F4/80 (green)/iNOS (red) or F4/80 (green)/Arg-1 (red) in each group of macrophages (scale bar: 50 μm, n = 5). **d–f** Representative immunoblots and quantification of macrophages in each group showing the expression of iNOS (**d, e**) and Arg-1 (**d, f**) after treatment for 24 h (n = 3). **g-h** The ratio of M1 macrophage phenotype-related surface markers, CD86/CD11b, were determined by flow cytometry for each group (n = 3). **i–j** The ratio of M2 macrophage phenotype-related surface markers, CD206/ CD11b, were determined by flow cytometry for each group (n = 3). In **a–j**, Error bars indicated the mean ± SD for three separate experiments. *P < 0.05, **P < 0.01, ***P < 0.001, and ****P < 0.0001. One-way ANOVA followed by Tukey's Post Hoc test

Immunofluorescence analyses were carried out to evaluate the polarization effect of Ang-(1–7) on macrophages in the simulated inflammatory environment in vitro. F4/80 is a typical marker of macrophages. iNOS and Arg-1 were used as M1 and M2 microglia/macrophages markers respectively (Fig. 2a). The results showed that the expression of Arg-1 in macrophages was significantly higher than that of control group after 24 h of Ang-(1–7) induction, while a few cells were found to express high levels of iNOS (not shown). In addition, after 24 h of LPS induction, the expression of iNOS in macrophages demonstrated a dramatic increase. Compared with the LPS group, the expression of iNOS in LPS + Ang-(1–7) group decreased (Fig. 2a, b). In addition, the expression level of Arg-1 in macrophages of LPS group was higher than that of control group, while the mean fluorescence intensity of Arg-1 expressed by macrophages in LPS + Ang-(1–7) group was the highest (Fig. 2a, c). This trend was verified again by Western Blot. Compared with the control group, there was a substantial increase in the expression of iNOS protein in LPS group and slight increase in the expression of Arg-1. The expression of iNOS in LPS + Ang-(1–7) group was decreased and the expression of Arg-1 was increased compared with LPS group (Fig. 2d–f). In addition, flow cytometry was used to characterize the polarization of macrophages. Compared with the LPS group, the proportion of CD86 + cells in the LPS + Ang-(1–7) group was decreased and the number of CD206 + cells was increased to a certain degree (Fig. 2 g–j). In summary, Ang -(1–7) can be applied to transform macrophage phenotype from M1 to M2.

### Ang-(1–7) regulates the expression of inflammatory cytokines in macrophages

**Fig. 3:**
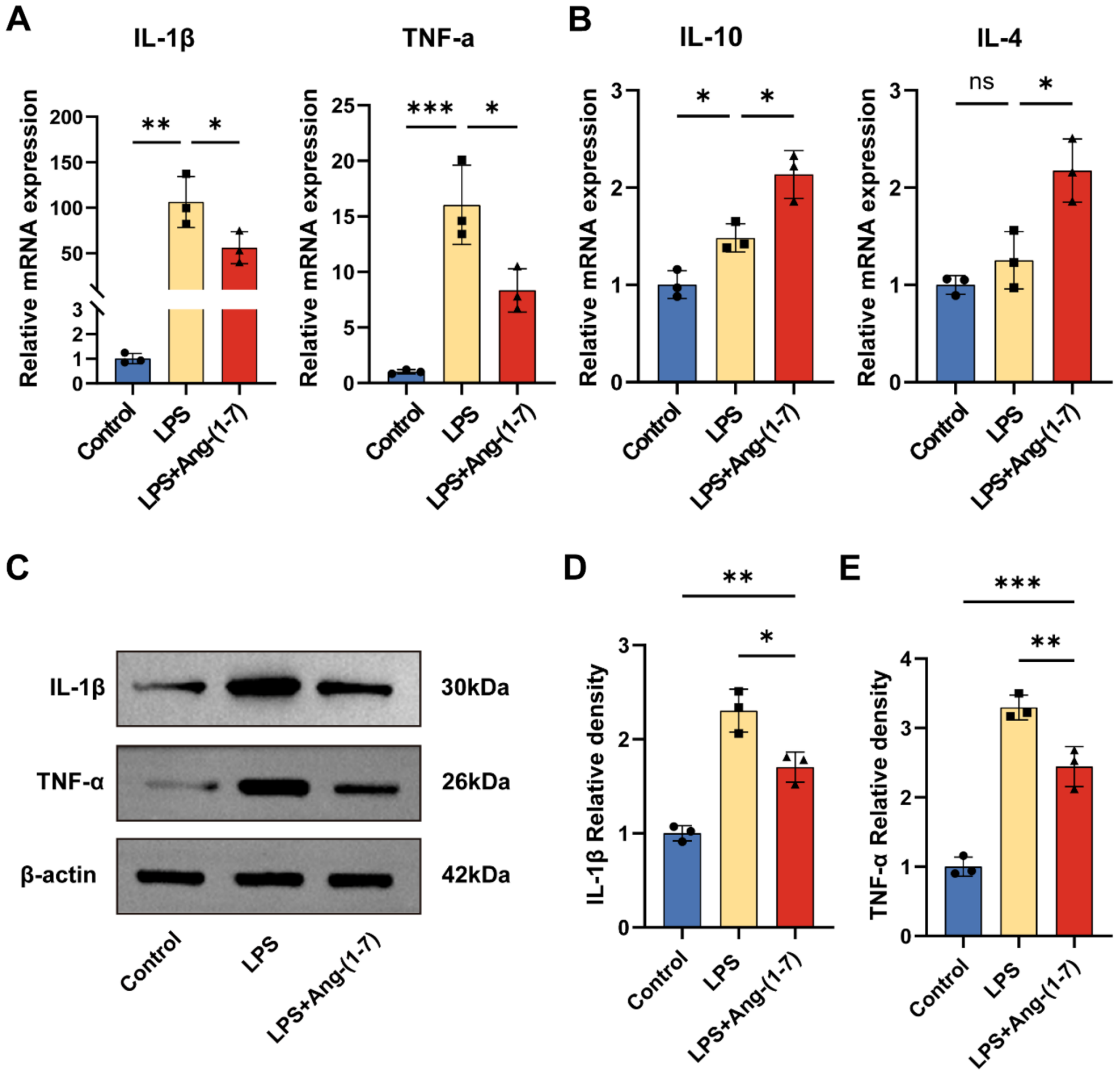
Ang-(1–7) inhibits inflammation response by modulating the expression of inflammatory cytokines. **a–b** Quantitative RT-qPCR analysis of macrophages showing mRNA expression of IL-1β, TNF-α (**a**), IL-10, IL-4 (**b**) after treatment for 24 h. GAPDH: loading control. Data are expressed as fold change compared to the control group (n = 3). **c–e** Representative immunoblots and quantification showing protein expression of IL-1β (**c, d**) and TNF-α (**c, e**) in the macrophages after treatment for 24 h. β-actin: loading control. Data are expressed as fold change compared to the control group (n = 3). In **a**–**e**, data are presented as mean ± SD; *ns* nonsignificant, P > 0.05; *P < 0.05, **P < 0.01, and***P < 0.001. One-way ANOVA followed by Tukey's Post Hoc test

To identify the regulatory role of Ang-(1–7) in producing inflammatory cytokines in BMDMs, we quantified the expression of inflammatory cytokine mRNA in different groups by RT-qPCR. After 24 h of LPS treatment, the expression levels of pro-inflammatory cytokines such as IL-1β and TNF-α were significantly increased. However, when macrophages were simultaneously treated with LPS and Ang-(1–7) for 24 h, the expression of IL-1β and TNF-α in macrophages were significantly reduced, while those of IL-4 and IL-10 were upregulated (Fig. 3a, b). Similarly, Western blot results showed that IL-1β and TNF-α protein expression levels were decreased in LPS + Ang-(1–7) group compared with the LPS group (Fig. 3c–e). These results suggest that Ang-(1–7) can inhibit the inflammatory response by regulating the expression level of BMDMs inflammatory cytokines.

### Local administration of Ang-(1–7) can improve functional recovery after spinal cord injury in rats

**Fig. 4:**
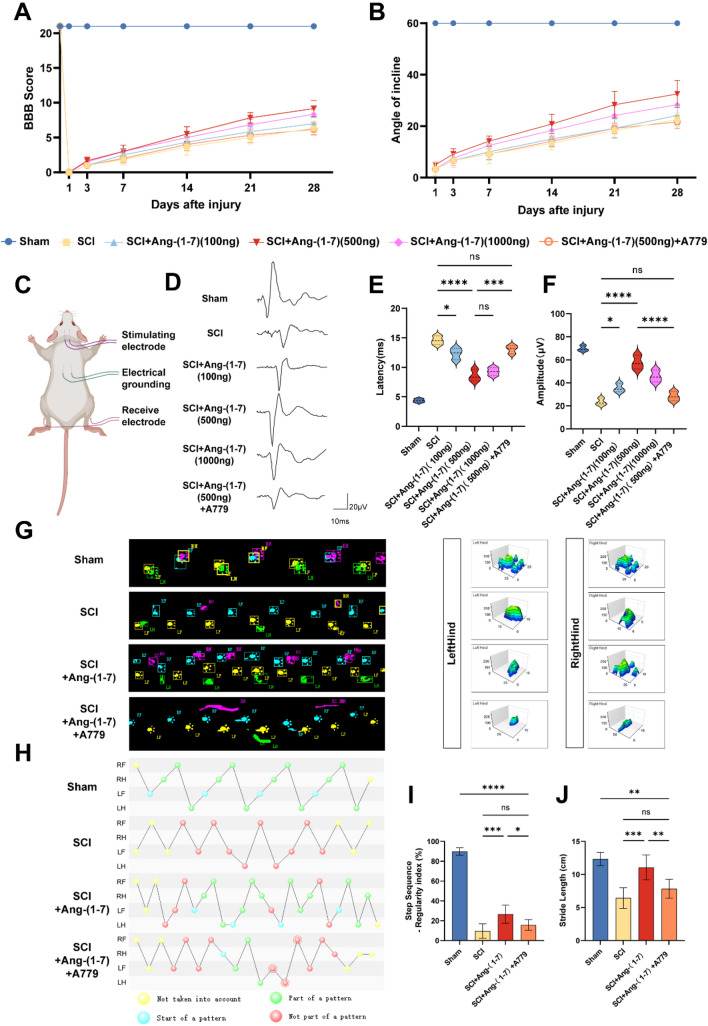
Ang-(1–7) improved the motor function of rats after SCI.** a** Basso, Beattie and Bresnahan (BBB) scores (n = 6). **b** Inclined plane test scores (n = 6). **c** Schematic diagram of electrophysiological motor evoked potential detection. **d–f** Motor-evoked potential (MEP) recordings quantification of Amplitude and Latency in each group on the 28th day post-surgery (n = 6). **g** Footprints and maximum contact area of rats in each group on the 28th day post-surgery (n = 6). **h** Footfall patterns of rats in each group on the 28th day post-surgery (n = 6). **i–j** Comparison of Regularity index and Stride length between each group of rats (n = 6). Error bars indicated the mean ± SD for three separate experiments. *ns* nonsignificant, P > 0.05; * P < 0.05, ** P < 0.01, *** P < 0.001, and **** P < 0.0001. In **a–b**, two-way ANOVA followed by Bonferroni’s post hoc tests. In **c-j**, one-way ANOVA followed by Tukey's Post Hoc test

To explore the role of Ang- (1–7) /MasR axis in regulating inflammation after SCI in rats, the range of drug dose was determined by the behavioral improvement effect, and A779 was used as a MasR antagonist to confirm whether Ang-(1–7)'s effect is through the MasR pathway. Throughout the recovery period within 4 weeks after SCI, BBB scores and inclined plane test suggested that the 500 ng Ang-(1–7) group had better functional recovery at 28 days after SCI, while the SCI + Ang-(1–7) + A779 group had a different degree of decrease in functional scores compared to the former. (Fig. 4a, b). MEP results showed that the amplitude of SCI group was significantly lower than that of Sham group on day 28 after surgery, and the latency was prolonged. It is worth noting that 500 ng Ang-(1–7) treatment can significantly enhance the MEP amplitude and shorten the latency; The amplitude of 1000 ng Ang-(1–7) treatment was slightly lower than that of the 500 ng Ang-(1–7) group, and the latency was not significantly different from that of the 500 ng Ang-(1–7) group. However, 100 ng Ang-(1–7) treatment only slightly improved MEP outcomes. Similarly, the application of A779 reversed the electrophysiological improvement of Ang-(1–7) in rats with SCI (Fig. 4c–f). Therefore, 500 ng Ang-(1–7) was selected as the standard dose for subsequent experiments. To better assess the motor function of the hind limbs in spinal cord injured rats, Catwalk footprint analysis was applied. The results indicated that the 500 ng Ang-(1–7) group showed better gait coordination (Fig. 4 g–i) and larger stride length (Fig. 4j) 28 days after surgery than the SCI group. In contrast, the hindlimb weight-bearing capacity of rats in the SCI + Ang- (1–7) + A779 group was weaker than that of the SCI + Ang- (1–7) group, and the improvement in gait coordination and stride length of the latter was reversed to varying degrees. These results suggest that activating the local Ang-(1–7) /MasR axis leads to better functional recovery in rats after SCI and that the MasR pathway plays an important role in the functional recovery produced by Ang-(1–7) action.

### Ang-(1–7) can improve histopathological results after spinal cord injury

**Fig. 5:**
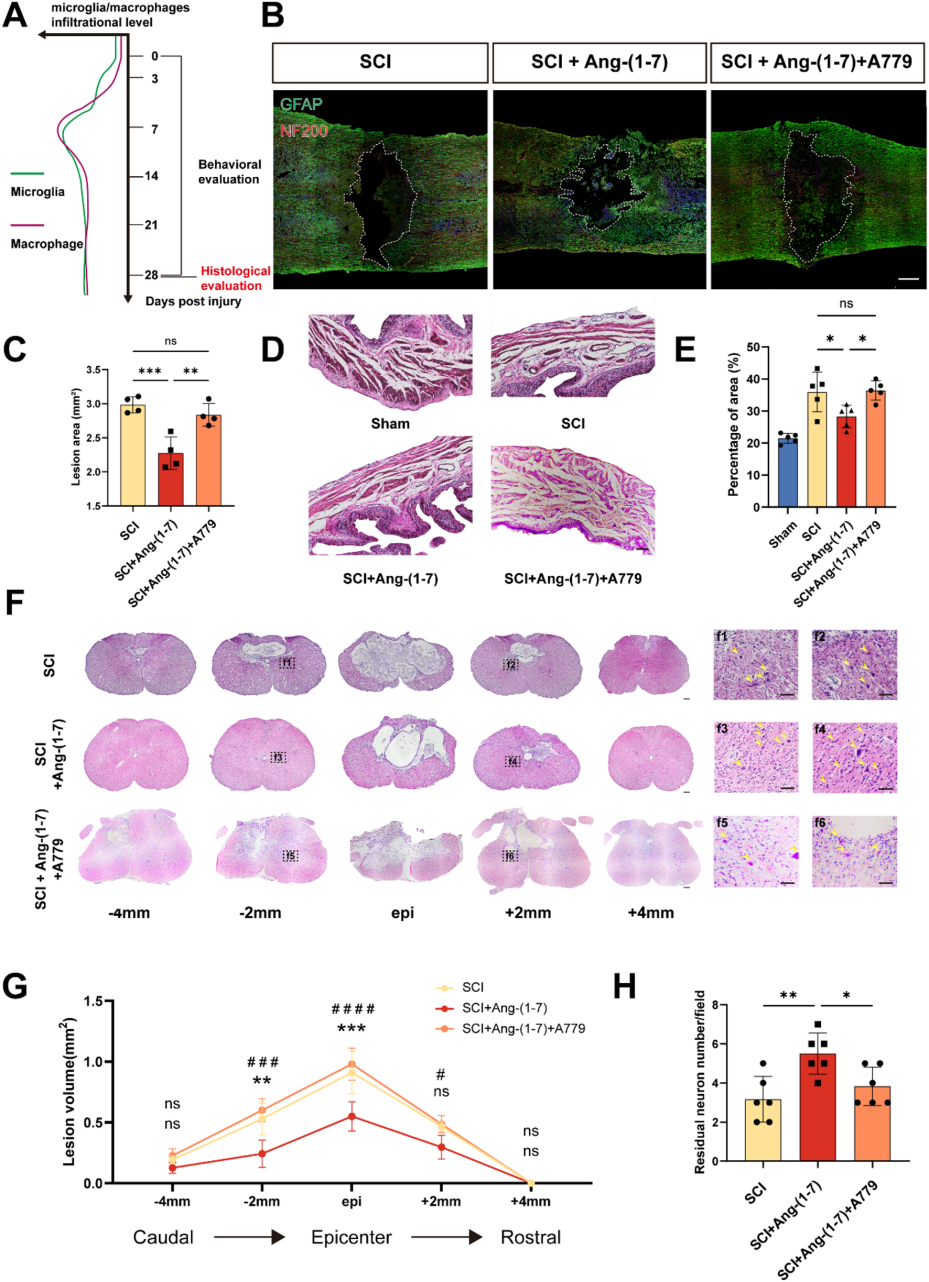
Ang-(1–7) improves histological results of spinal cord and bladder after SCI. **a** Microglial macrophage infiltration curves and time axis of histological and functional evaluation after SCI. **b–c** Double-staining for GFAP (green)/NF200 (red) and quantification of spinal cord sections in each group on the 28th day post-surgery. (scale bar: 500 μm, n = 4). Dashed line area shows cavity of spinal cord. **d–e** HE staining and quantification of bladder on the 28th day post-surgery. (scale bar: 100 μm, n = 5). **f** Continuous sections of the spinal cord by HE staining were obtained on the 28th day post-surgery, and the remaining neurons in the SCI group (**f1, f2**), SCI + Ang-(1–7) groups (**f3, f4**), and SCI + Ang-(1–7) + A779 groups (**f5, f6)** at the same distance from the epicenter were displayed. (scale bar: 100 μm and 40 μm, n = 3). Arrows indicate the remaining neurons. **g** Comparison of spinal cavity area at different sections. **h** Counting analysis of ventral neurons at rostral 2 mm and caudal 2 mm. (n = 5). *ns* nonsignificant, P > 0.05; *, #P < 0.05, **P < 0.01; ***, ###P < 0.001; and ####P < 0.0001. In **a–e**, **h** one-way ANOVA followed by Tukey’s Post Hoc test. In **g**, two-way ANOVA followed by Bonferroni’s post hoc tests

Microglia/macrophage infiltration can aggravate secondary injury, and parenchymal cell death in inflammatory microenvironment, combined with phagocytosis, leads to syringomyelia. The histological analysis of the spinal cord and bladder 28 days after surgery was used to evaluate the severity of secondary injury in each group (Fig. 5a). The nerve fibers and astrocytes labeled by NF200 and GFAP respectively to the visualization of spinal cord morphology. The rats in groups of SCI and SCI + Ang-(1–7) + A779 presented severe nerve fiber fracture and spinal cavity, while the SCI + Ang-(1–7) group showed weaker lesions at similar sites, indicating the protective effect of Ang-(1–7) on secondary injury after SCI (Fig. 5b, c). In addition, the repair effect of Ang-(1–7) on SCI was evaluated by comparing the HE staining of bladder tissues in each group. HE staining revealed that the collagen content of the bladder wall was significantly more in the SCI group than in the Sham group, indicating that the bladder tissue was compensated under continuous high pressure, and the bladder collagen layer in the SCI + Ang-(1–7) group was improved compared with that in the SCI group. In contrast, the proportion of collagen fibers to the total area in the bladder wall was significantly higher in the Ang-(1–7) + A779 group compared to the Ang-(1–7) group (Fig. 5d, e). Similarly, the size of the cavity and the number of residual neurons were identified by HE staining of cross-sections of the spinal cord. Compared with SCI group, SCI + Ang-(1–7) group displayed a smaller lesion area and more residual neurons in the lesion center and the rostral and caudal sides, but this improvement was reversed in the SCI + Ang-(1–7) + A779 group (Fig. 5f–h). The data suggest that early activation of the Ang-(1–7)/MasR pathway after SCI could improve histological outcomes.

### Local injection of Ang-(1–7) promoted M2 polarization of macrophages/microglia

**Fig. 6:**
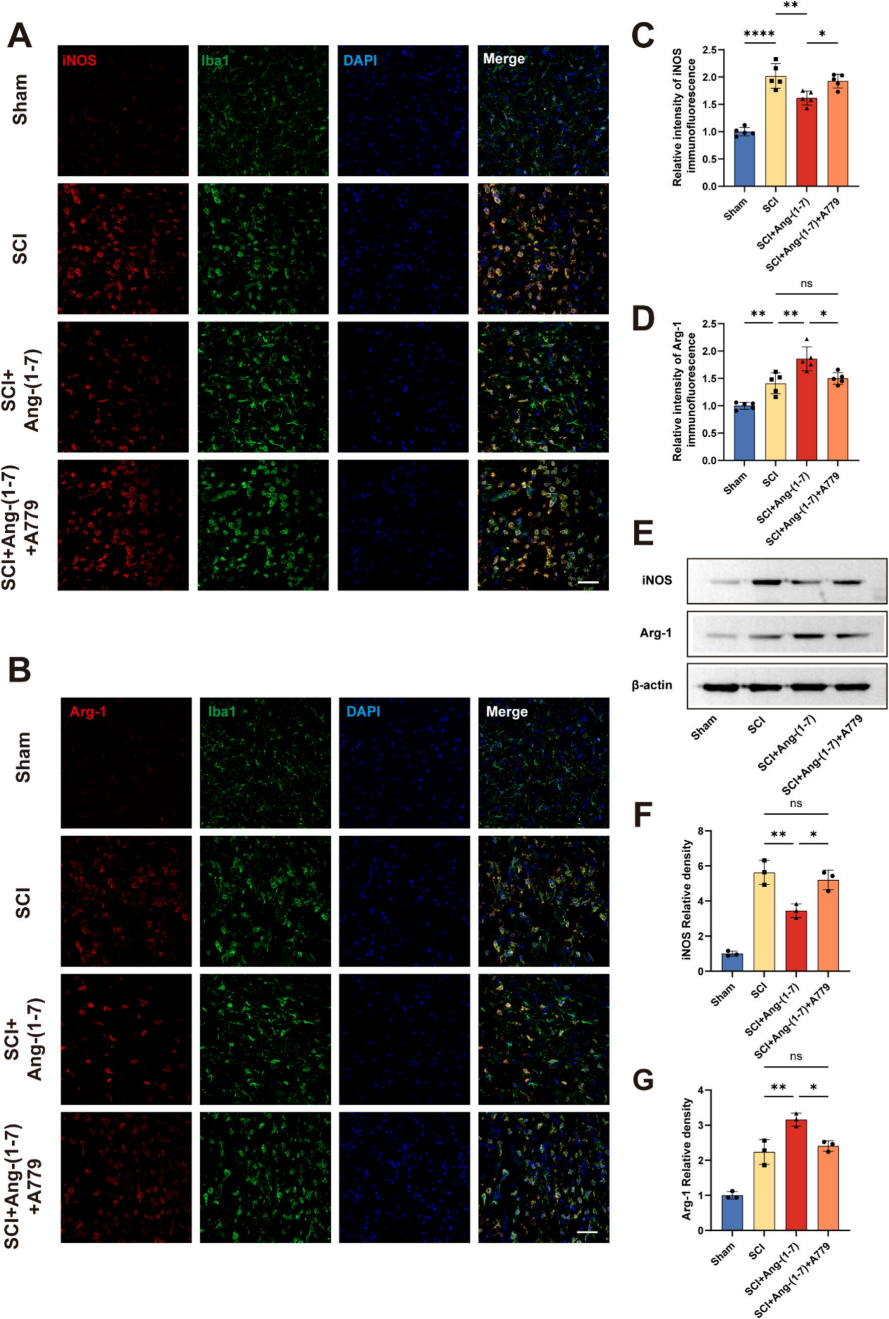
Ang-(1–7) shifts the polarization of microglia/macrophages in the spinal cord toward the M2 phenotype **a-b** Double-staining for Iba1 (green)/iNOS (red) and Iba1 (green)/Arg-1 (red) of spinal cord tissue sections in each group on the 3rd day post-surgery (scale bar: 50 μm, n = 5). **c-d** quantification of spinal cord tissue sections in each group on the 3rd day post-surgery (scale bar: 50 μm, n = 5). **e–g** Representative immunoblots and quantification of iNOS (**e, f**) and Arg-1 (**e, g**) in the spinal cord tissue in each group on the 3rd day post-surgery. Data are expressed as fold change compared to the control group (n = 3). In **a–g**, data are presented as mean ± SD; *P < 0.05, **P < 0.01, and ****P < 0.0001. One-way ANOVA followed by Tukey's Post Hoc test

M1-type microglia/macrophages in the acute stage after spinal cord injury can continuously release inflammatory chemotactic mediators and pro-inflammatory cytokines, causing great pressure on the survival of neurons [7, 25]. Spinal cord tissues collected 3 days post-surgery were stained with iNOS/Iba1 or Arg-1/Iba1 to evaluate the polarization of microglia/macrophages. It was noted that the average fluorescence intensity of iNOS in Iba1 + cells in SCI + Ang-(1–7) group was lower than that in SCI group, while the expression of Arg-1 was opposite. In addition, iNOS expression was higher in the SCI + Ang-(1–7) + A779 group of Iba1 + cells than in the SCI + Ang-(1–7) group, whereas Arg-1 expression was lower (Fig. 6a–d). These findings suggest that local intrathecal injection of Ang-(1–7) after SCI can reduce the expression of iNOS in Iba1+ cells and increase the expression of Arg-1. More microglia/macrophages were promoted to convert to the M2 phenotype after spinal cord injury, and this effect was attenuated after MasR was antagonized.

In addition, Western blot was adopted to further detect the changes in iNOS and Arg-1 protein level in groups SCI and SCI + Ang-(1–7). Consistent with the results of vitro study, SCI + Ang-(1–7) group reduces the expression of iNOS and increases the expression of Arg-1. Similarly, compared with the SCI + Ang-(1–7) group, the SCI + Ang-(1–7) + A779 group showed higher iNOS expression and lower Arg-1 protein level (Fig. 6e–g). To sum up, these results suggest that Ang-(1–7) treatment can promote the polarization of microglia/macrophages to the M2 phenotype and enhance the shift from pro-inflammatory responses to anti-inflammatory responses, and this effect can be partially blocked by A779.

### Ang-(1–7) reduces CD68 + cell infiltration and regulates inflammatory cytokine expression after SCI

**Fig. 7:**
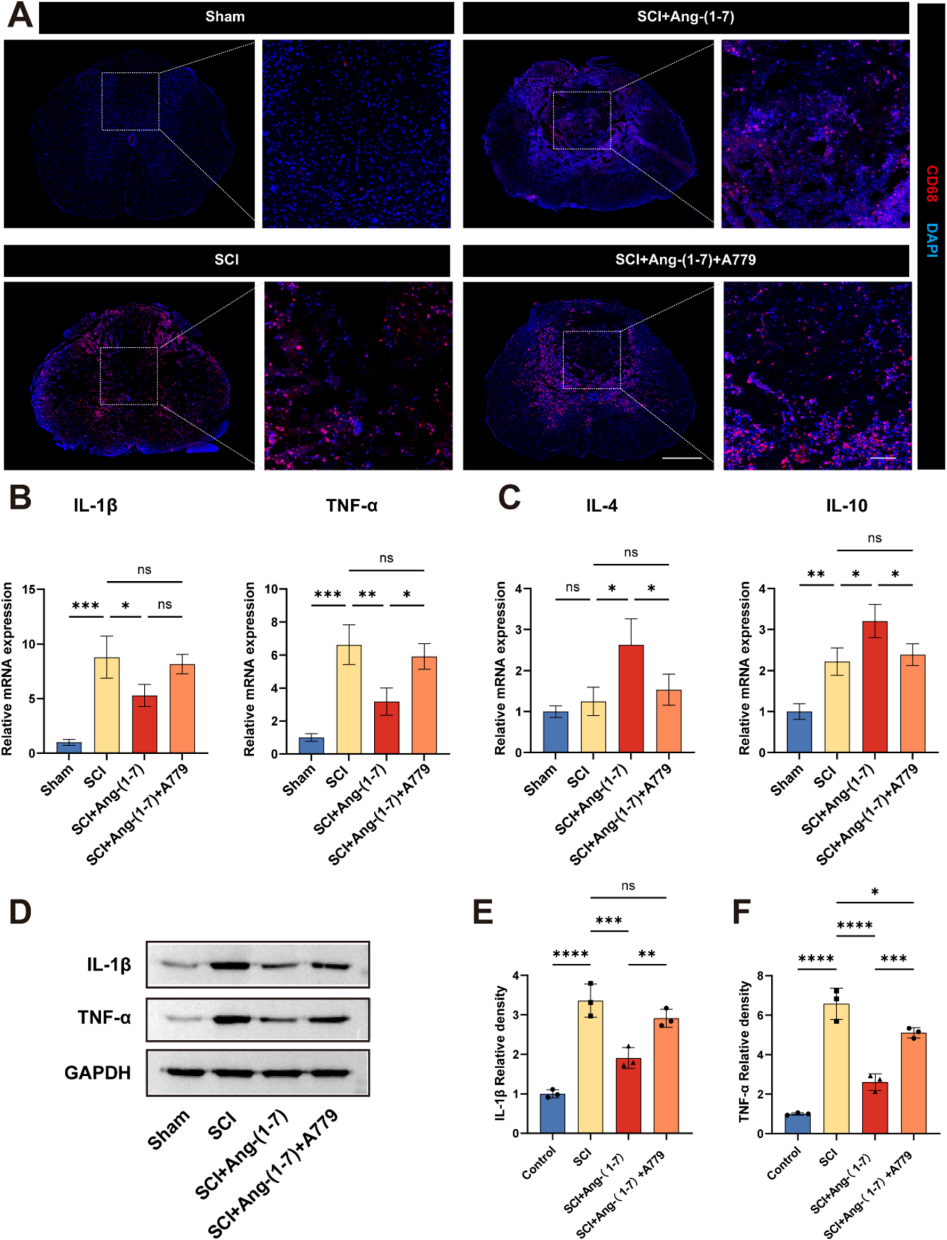
Ang-(1–7) reduced inflammatory cell infiltration and regulated inflammatory cytokines in SCI rats. **a** Representative fluorescence image of the distribution of CD68^+^ cells in each group of spinal cord sections (scale bar: 500 μm and 100 μm, n = 5). **b–c** Quantitative RT-qPCR analysis of spinal cord tissue showing mRNA expression of IL-1β, TNF-α (**b**), IL-10, IL-4 (**c**) on the 3rd day post-surgery. Data are expressed as fold change compared to the control group (n = 3). **d**–**f** Representative immunoblots and quantification showing protein expression of IL-1β (**d, e**) and TNF-α (**d, f**) in the spinal cord tissue on the 3rd day post-surgery. GAPDH: loading control. Data are expressed as fold change compared to the control group (n = 3). In a–f, data are presented as mean ± SD; *P < 0.05, **P < 0.01, ***P < 0.001, and ****P < 0.0001. One-way ANOVA followed by Tukey’s Post Hoc test

Next, we attempted to observe whether intrathecal injection of Ang-(1–7) after spinal cord injury could inhibit local inflammatory response. CD68, one of the specific markers of activated phagocytes, was used in this study to evaluate injured spinal cord tissue sections. CD68 showed low expression in normal spinal cord tissue and the number of CD68 + cells in the SCI + Ang-(1–7) group was fewer than that in the SCI group. In addition, the number of CD68 + cells in the SCI + Ang-(1–7) + A779 group was significantly higher than that in the SCI + Ang-(1–7) group (Fig. 7a). In addition, RT-qPCR analysis of spinal cord tissue 3 days post-surgery showed that local administration of Ang-(1–7) after SCI down-regulated the expression of pro-inflammatory cytokines such as IL-1β and TNF-α, and up-regulated the expression of anti-inflammatory cytokines such as IL-4 and IL-10, but this trend was reversed in the SCI + Ang-(1–7) + A779 group (Fig. 7b, c). Western blotting results of spinal cord tissue 3 days post-surgery showed that the expression levels of IL-1β and TNF-α in SCI + Ang-(1–7) group were lower than those in the SCI group and the SCI + Ang-(1–7) + A779 group (Fig. 7d–f).

### MasR plays a part in SCI recovery

**Fig. 8:**
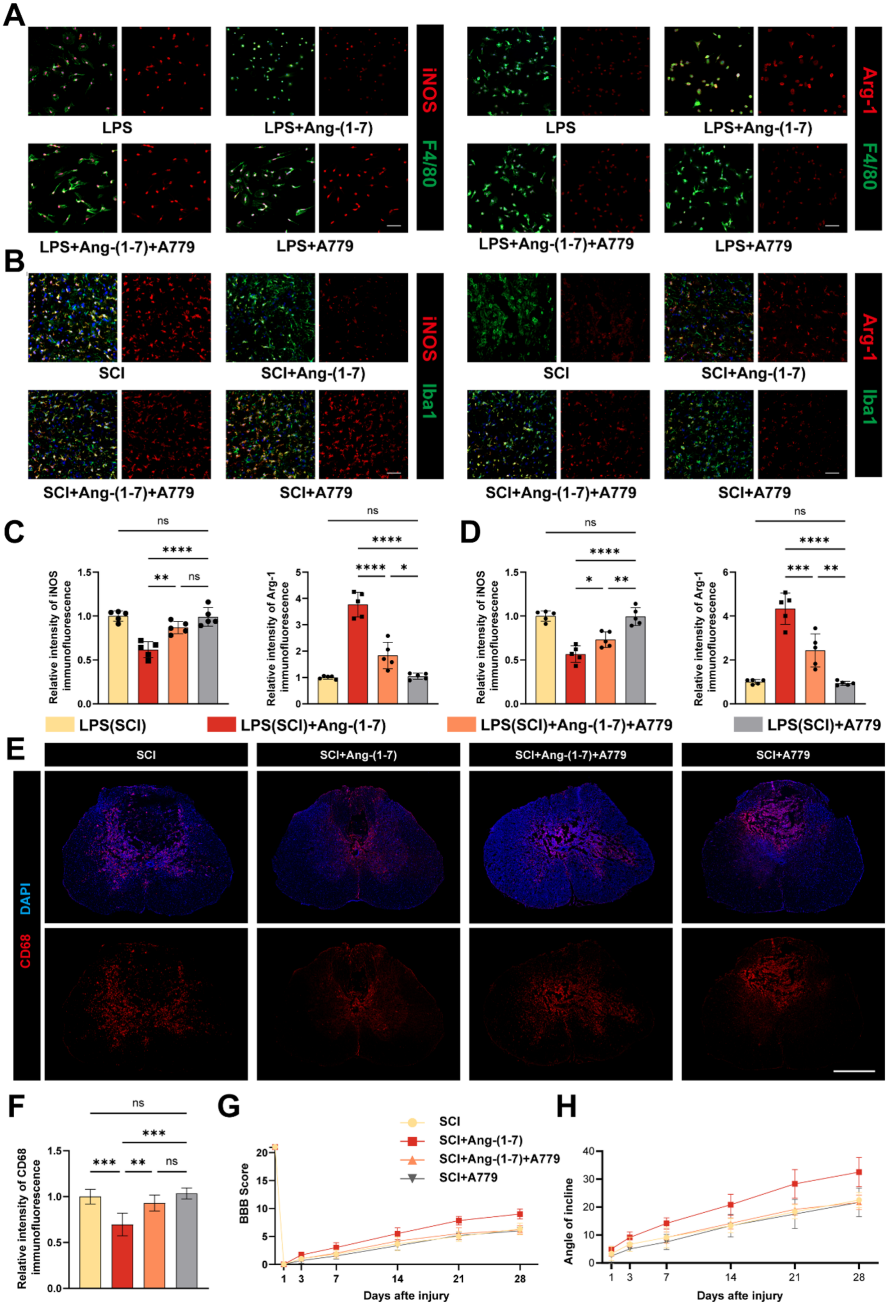
MasR mediates Ang-(1–7) inhibition of inflammation and improvement in promoting neurological recovery. **a** Double-staining for F4/80 (green)/iNOS (red) or F4/80 (green)/Arg-1 (red) in each group of macrophages (scale bar: 50 μm, n = 5). b Double-staining for Iba1 (green)/iNOS (red) or F4/80 (green)/Arg-1 (red) of spinal cord sections in each group on the 3rd day post-surgery (scale bar: 50 μm, n = 5). **c**–**d** Quantification of iNOS and Arg-1 immunofluorescence intensity in vivo and in vitro for each group of microglia/macrophages. **e** Representative fluorescence image of the distribution of CD68^+^ cells in the group of SCI, SCI + Ang-(1–7), SCI + Ang-(1–7) + A779 and SCI + A779 (scale bar: 500 μm, n = 3). **f** Quantification of CD68 immunofluorescence intensity in spinal cord tissue sections from each group. **g** Basso, Beattie and Bresnahan (BBB) scores. **h** Inclined plane test scores. Data are presented as mean ± SD; ns, nonsignificant, P > 0.05; *P < 0.05, **P < 0.01, ***P < 0.001, and**** P < 0.0001. In **a**–**f**, one-way ANOVA followed by Tukey's Post Hoc test. In **g**–**h**, two-way ANOVA followed by Bonferroni’s post hoc tests

We investigated further the role of MasR in regulating inflammation and its impact on recovery from spinal cord injury. Immunofluorescence staining of microglia/macrophages polarization in vitro and in vivo displayed that the regulatory effect of Ang-(1–7) on microglia/macrophages in the inflammatory environment was reversed by A779. In addition, no significant difference in microglia/macrophage phenotype between the LPS (SCI) and LPS + A779 (SCI + A779) groups (Fig. 8a–d) were found in immunofluorescence results. Moreover, we found that the distribution number of CD68 + cells in the SCI + Ang-(1–7) + A779 group was higher than that in SCI + Ang-(1–7) group, suggesting that the effect of Ang-(1–7) on alleviating inflammatory cell infiltration was also partially blocked by A779, and the number of CD68 + cells was the highest in the SCI and SCI + A779 groups, with no significant difference between the two groups (Fig. 8e, f). BBB score and oblique plate test results 4 weeks after surgery showed that the recovery of the SCI group, the SCI + Ang-(1–7) + A779 group, and the SCI + A779 group were worse than that of the SCI + Ang-(1–7) group, and no significant difference in recovery between the three groups was found, indicating that MasR played an important role in SCI recovery (Fig. 8f–h). These combined data suggest that activation of Ang-(1–7)/MasR Axis is conducive to regulate microglia/macrophages polarization, inflammatory cell infiltration, and functional recovery of spinal cord injury. In addition, antagonism of MasR after SCI does not lead to worse prognosis.

### Ang-(1–7)/MasR axis regulates inflammatory responses after SCI through TLR4-mediated NF-κB pathways

**Fig. 9:**
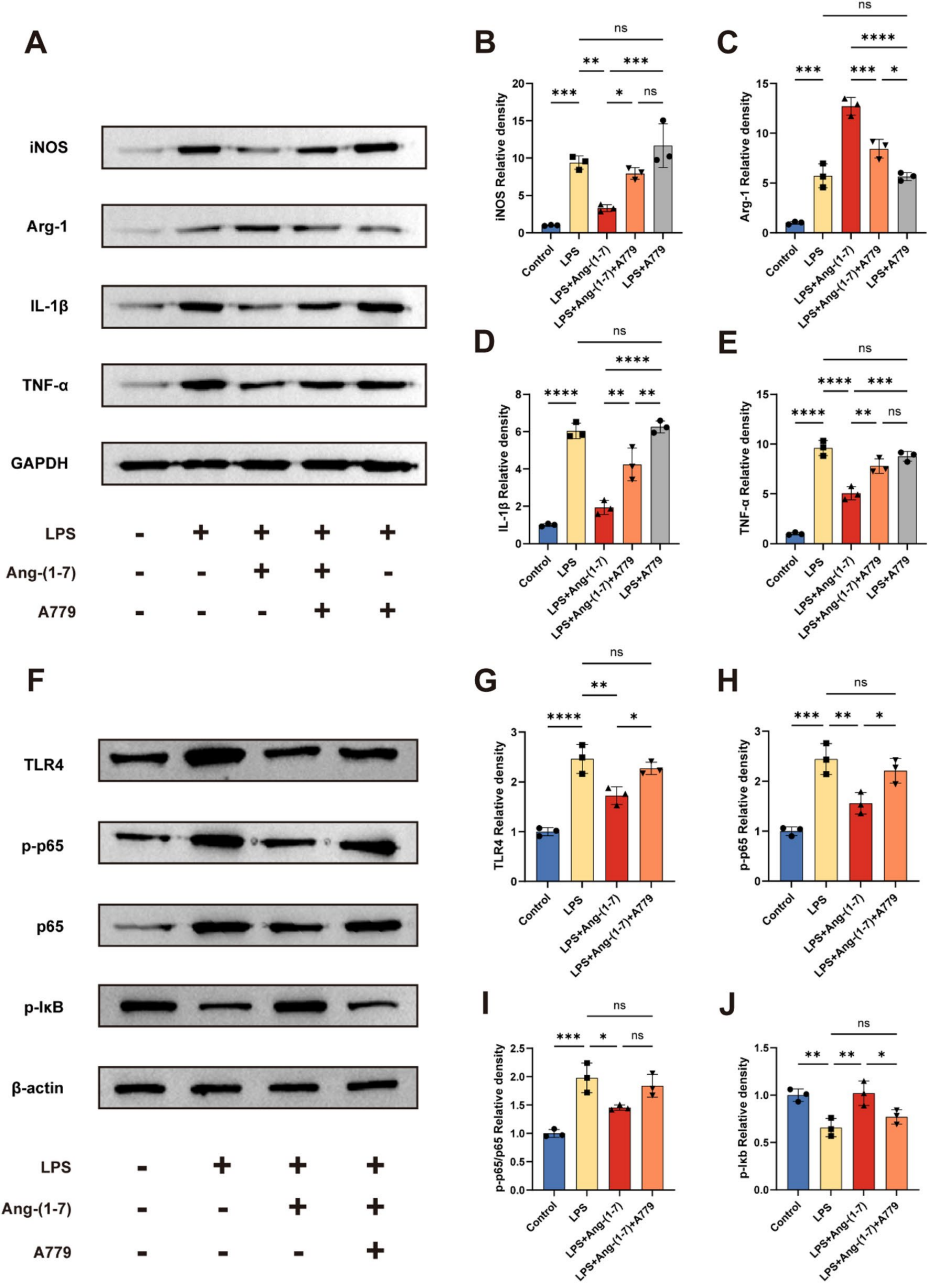
Ang-(1–7) regulates microglia/macrophage polarization and inflammatory cytokine secretion through the TLR4-mediated NF-κB pathway. **a** Representative Western blots for iNOS, Arg-1, IL-1β, TNF-α and GAPDH expression in each group rats. **b**–**e** Bar graph showing the corresponding quantitative data. **f** Representative Western blots for TLR4, p-p65, p65, p-Iκb and β-actin expression in each group rats. The TLR4/ NF-κB pathway was inhibited by Ang-(1–7) treatment in the indicated group and A779 partially reversed the effects of Ang-(1–7). **g**–**j** Bar graph showing quantitative data for each group of NF-κB-related proteins. Error bars indicated the mean ± SD for three separate experiments, n = 3 for each group, ns, nonsignificant, P > 0.05; *P < 0.05, **P < 0.01, ***P < 0.001, and ****P < 0.0001. one-way ANOVA followed by Tukey's Post Hoc test

The TLR4/NF-κB pathway plays an essential role in activating and polarizing microglia/macrophage [26, 27]. Here, to explore further the possible mechanism by which Ang-(1–7)/MasR Axis regulates inflammatory response to repair spinal cord injury, we investigated whether TLR4/NF-κB pathway is involved in this process. WB analysis showed that the expression of iNOS, IL-1β, and TNF-α was significantly increased in LPS-induced macrophages, but macrophages intervened by LPS in combination with Ang-(1–7) were able to reverse the increase in the expression of these proteins, as well as upregulate the expression of Arg-1. In addition, A779 was able to partially inhibit the regulation of these proteins by Ang-(1–7). Notably, there was no significant difference in protein expression between the LPS + A779 and LPS groups, suggesting an essential role of MasR activation in the regulation of macrophage phenotype (Fig. 9a–e). Therefore, we focused on the effect of activation of the Ang-(1–7)/MasR axis on key proteins of the TLR4/NF-κB pathway. the LPS + Ang-(1–7) group significantly reduced LPS-induced TLR4, p-p65/p65 upregulation, and upregulated IκB expression. Similarly, A779 partially reversed the effect of Ang-(1–7) (Fig. 9f–j). It is suggested that the Ang-(1–7)/MasR axis alleviates inflammatory response and promotes functional recovery after spinal cord injury at least partly through the TLR4-mediated NF-κB signaling pathway.

## Discussion

As a serious central nervous system disease, spinal cord injury can cause acute neurological deficits below the injury plane and other symptoms, including chronic neuralgia [28]. SCI therapy is challenging worldwide due to the insufficient regenerative capacity of the central nervous system and the existence of local inhibitory microenvironment [29]. Among them, inflammatory response after spinal cord injury is the most significant part in the pathological process of spinal cord injury, which is typically manifested by the activation of microglia/macrophages and changes of local inflammatory factors after spinal cord injury [30]. Neuroinflammatory regulation is considered to be an important strategy for repairing SCI by alleviating secondary injury [31]. In this study, we demonstrate that activation of the Ang-(1–7)/MasR axis improves histological and functional outcomes in SCI rats by regulating M1/M2 polarization of microglia/macrophages and inflammatory cytokines. MasR antagonist A779 significantly inhibited M2-like polarization of microglia/macrophages by Ang-(1–7) and increased M1-like polarization, thereby exacerbating secondary spinal cord injury and functional impairment after SCI. Mechanistically, Ang-(1–7)/MasR axis mediates the TLR4/NF-κB signaling pathway to regulate microglia/macrophage polarization.

Microglia, as resident immune cells of the nervous system, can react and migrate to the site of injury within minutes after SCI [32, 33]. In addition, due to the destruction of the blood spinal cord barrier and the secretion of a large number of inflammatory cytokines, blood monocytes infiltrate into the site of spinal cord injury and differentiate into macrophages, and polarization occurs under the stimulation of the local inflammatory microenvironment [34]. This process can reach its peak 7 days after spinal cord injury [35]. In the acute stage after spinal cord injury, M1 microglia/macrophages are a dominant player. Due to the up-regulated expression of intracellular NADPH enzyme, high levels of oxidative metabolites (such as superoxide and nitric oxide) may cause collateral damage to normal tissues [36]. In addition, M1 microglia/macrophages release large quantities of pro-inflammatory mediators and inflammatory cytokines combined with chondroitin sulfate proteoglycan expression to promote neuronal necrosis and inhibit its growth [7, 37]. Moreover, these activated inflammatory cells can remain in the infiltrating site for a long time and maintain inflammation, which adversely affects local nerve regeneration [38]. While M2 microglia/macrophages have been shown to be beneficial in the treatment of non-infectious CNS inflammation including SCI [39, 40]. It has also been reported that M2 microglia/macrophages can enhance phagocytosis, and arginase-1 expressed by M2 microglia/macrophages can antagonize iNOS and contribute to anti-inflammatory reaction [41]. Yan-qing Wu et al. proved that metformin can advance the transformation of microglia M1–M2 phenotype polarization, thus greatly promoting the clearance of myelin fragments and protecting myelin sheath in SCI rats [42]. Guodong Sun et al. demonstrated that human umbilical cord mesenchymal stem cell exosomes can effectively trigger BMDM phenotype to M2 polarization and improve functional recovery after SCI by down-regulating pro-inflammatory cytokines [43]. Multimolecular pathways such as nuclear factor -κB (NF-κB), MAPK, interferon regulatory factor (IRF), and STAT are involved in mitigating M1 and M2 phenotypes [44–48]. In this study, we suggest that Ang-(1–7)/MasR axis was more likely to be activated under inflammatory conditions. Furthermore, Immunofluorescence, Western Blot and flow cytometry results showed that Ang-(1–7) could advance the transformation of microglia/macrophages from M1 phenotype to M2 phenotype. Similarly, RT-qPCR results clarified that pro-inflammatory cytokines were down-regulated and anti-inflammatory cytokines were up-regulated in Ang-(1–7) group.

CD68, a lysosomal membrane-associated protein, is detected at high levels mainly in innate immune cells of activated phagocytes, but has limited expression in tissue-resident macrophages and microglia [49, 50]. Similarly, as shown in our results, CD68 was lowly expressed in the spinal cord of the Sham group, while the number of CD68 + cells in the spinal cord of the SCI group was significantly increased compared to the former. Activated microglia/macrophages are considered to have more detrimental than beneficial effects in secondary spinal cord injury, even though CD68 + cells contribute to the clearance of cellular debris after SCI. In studies where MSCs, drugs, and tissue material inhibit microglia activation after spinal cord injury, a decrease in CD68 + cells is thought to be associated with enhanced tissue preservation and eventual functional recovery [51–53]. Previous results showed that the area of spinal cord injury in Ang-(1–7) group was reduced, the number of remaining neurons was increased, and bladder function was improved to some extent. In motor function, compared with SCI group, the BBB score and inclined plate test score of Ang-(1–7) group were higher, the MEP results were improved, and the motor coordination and stride were recovered to a certain extent. These results indicate that Ang-(1–7) treatment can effectively alleviate the secondary injury after SCI and promote the recovery of histological and motor function in rats by promoting the transformation of microglia/macrophages to M2 phenotype and regulating the expression of inflammatory cytokines.

Ang-(1–7), a fragment of Ang-II produced by ACE2 action, is considered to be a bioactive end product of RAS. Mainly mediated by MasR, Ang-(1–7) is has the opposite effect to classical RAS [15, 54]. It has been verified by a handful number of reports that Ang-(1–7) can regulate inflammation and alleviate oxidative stress, and ischemia–reperfusion in the local environment of brain, kidney, heart, liver, lung, and joints [20, 55–58]. Recent clinical longitudinal studies have shown that Ang-(1–7) is locally upregulated after mild traumatic brain injury in humans, and that Ang-(1–7) gradually decreases as inflammation subsides in mTBI patients: this can be viewed as an attempt to balance pro-inflammatory and anti-inflammatory events [25]. Research by Ruili Dang et al. showed that Activation of the ACE2/Ang(1–7)/MasR axis triggers the Forkhead box Class O1 (FOXO1) -autophagy pathway and induces FoxO1-targeted superoxide dismutase (SOD) and catalase (CAT) of microglia for neuroprotective effects [26]. Therefore, we attempted to verify whether Ang-(1–7) has a repairing effect on traumatic spinal cord injury. Immunofluorescence staining of spinal cord tissue sections collected on the 3th day after surgery showed that the expression of Arg-1 was up-regulated and that of iNOS was down-regulated in infiltrated microglia/macrophages compared with SCI group. These results indicate that Ang-(1–7) can regulate the polarization of microgel/macrophage towards M2 in vivo, which are consistent with the results in vitro. In addition, the number of CD68 + cells in the Ang-(1–7) group was significantly lower than that in the SCI group, suggesting that Ang-(1–7) could reduce inflammatory cell infiltration after SCI. Histological results showed that the Ang-(1–7) group had less lesion area, preserved more neurons, and improved bladder function compared with the SCI group. The Locomotor analysis indicated that Ang-(1–7) group showed better recovery of hind limb function than SCI group.

The role of MasR was also researched in this study. Similar to the study conducted by Isabella Zaidan et al., LPS contributes to the upregulation of MasR expression [59]. Furthermore, our results suggest that the action of Ang-(1–7) is equally able to upregulate the expression of MasR in macrophages. Immunofluorescence results showed that the expression of Arg-1 was significantly higher than that of the control group after 24 h of Ang-(1–7) action on macrophages, while most macrophages expressed lower levels of iNOS, similar to the control group, but a small number of cells expressed high levels of iNOS. This indicated that the action of Ang-(1–7) contributed to the M2-like phenotype of most macrophages, while some cells exhibited elevated expression of both iNOS and Arg-1. In addition, LPS-induced MasR was also upregulated in M1-type macrophages, indicating that the upregulation of MasR may be related to the state of macrophage activation and less to the specific phenotype. Moreover, the effect of Ang-(1–7) was greatly inhibited by the application of A779 to antagonize MasR. However, in the LPS + A779 (SCI + A779) group, the phenotype of microglia/macrophages was not significantly different from that of the LPS (SCI) group. This demonstrates the significant role of Ang-(1–7)/ MasR axis in regulating microglia/macrophage polarization, but antagonization of MasR may have little effect on microglia/macrophage phenotype.

An increasing amount of data suggest that TLR4/NF-κB pathways engage in regulating microglia/macrophage polarization. To improve understanding in the relationship between Ang-(1–7)/MasR axis and TLR4/NF-κB pathway, Western blot was used to detect the expression changes of key proteins in the pathway after activation and blockade of MasR. We found that Ang-(1–7) inhibited the expression of LPS-stimulated TLR4/NF-κB, while the pretreatment of A779 partially reversed the inhibitory effect of Ang-(1–7). These results suggest that the activation of Ang-(1–7)/MasR axis regulates microglia/macrophage polarization at least in part through the TLR4/NF-κB pathway. However, further experiments are needed to determine whether other pathways mediate Ang-(1–7) to regulate microglia/macrophage polarization.

Although our results show that Ang-(1–7) has a conducive effect on SCI repair by modulating the inflammatory response, the behavioral improvement of Ang-(1–7) after SCI in rats still lags behind. Given the complex mechanism of secondary spinal cord injury, immunomodulation alone may not achieve satisfactory therapeutic efficacy, but can be used as an alternative to clinical combination therapy. Since infiltration of peripheral monocytes peaks as early as 7 days after spinal cord injury, early Ang-(1–7) intervention may be more helpful in modulating the immune microenvironment and improving the prognosis of spinal cord injury. In addition, subdural injections are more likely to be applied clinically because of their ability to avoid systemic reactions and to act more quickly on the site of injury.

Although we focused on the potential role of the Ang-(1–7)/MasR axis in regulating microglia/macrophage polarization for SCI repair, the current study still has some limitations. (1) A rising number of studies have shown that ACE is also essential to regulate adaptive immunity and that adaptive immunity is also of great importance. The current study focuses on the role of Ang-(1–7)/MasR axis in regulating innate immunity after spinal cord injury, while the Ang-(1–7)/MasR axis fails to consider the role of adaptive immunity. (2) We cannot rule out the possibility that Ang-(1–7) has a direct protective effect on neurons and the blood-spinal barrier [20]. (3) The Spatio-temporal spectrum of local Ang-(1–7) expression after SCI and the exact mechanism of Ang-(1–7) intervention on microglia/macrophages need to be further explored in future studies.

## Conclusions

**Fig. 10:**
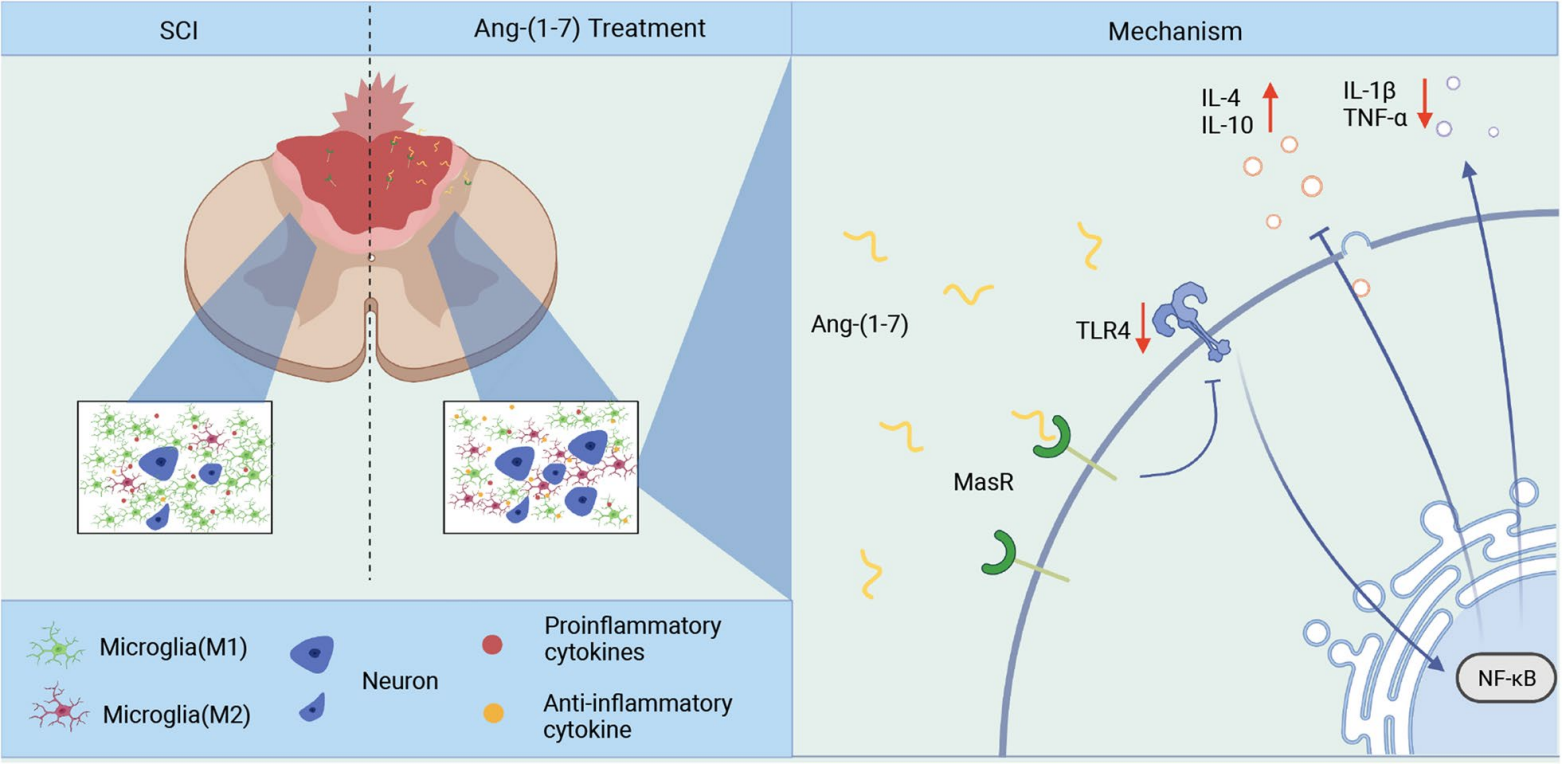
Schematic diagram of inflammatory regulation and neuroprotective role of Ang-(1–7)/MasR axis in spinal cord injury

In summary, our findings indicated that activating Ang-(1–7)/MasR axis can significantly regulate microglia/macrophage polarization, reduce inflammatory cell infiltration, alleviate secondary injury and promote motor function recovery in SCI rats, which was partly dependent on TLR4/NF-κB pathway. The current study revealed the anti-inflammation property of Ang-(1–7) and its underlying mechanism and provided novel therapeutic strategies for treating spinal cord injury (Fig. 10).

## Data Availability

All data supporting the conclusions of this manuscript are provided in the text and figures.

## Abbreviations

SCI: Spinal cord injury
LPS: Lipopolysaccharide
DMEM: Dulbecco’s modified Eagle’s medium
PBS: Phosphate-buffered solution
ACE2: Angiotensin-converting enzyme 2
DAPI: 4',6-Diamidino-2-phenylindole
TBST: Tris-buffered saline with Tween
PBST: Phosphate-buffered saline with Tween
WB: Western Blot
HE: Hematoxylin–eosin staining
ANOVA: Analysis of variance
BBB: Basso, beatlie, bresnahan
iNOS: Inducible nitric oxide synthase
Arg-1: Arginase-1
IL-1β: Interleukin-1β
TNF-α: Tumor necrosis factor-α
TLR4: Toll like receptor 4
NF-κB: Nuclear factor κB
IκB: NF-κB inhibitor
GAPDH: Glyceraldehyde-3-phosphate dehydrogenase
Iba1: Ionized calcium-binding adapter molecule 1
